# Isolation and Pathogenicity Analysis of a Novel Orthoreovirus Caused the Outbreak of Duck Viral Arthritis in China

**DOI:** 10.1155/2023/8179312

**Published:** 2023-05-10

**Authors:** Bing Li, Xiaoning Jiang, Shuai Zhang, Qianqian Wang, Yitong Cui, Qiong Wu, Wensha Jia, Jie Zhang, Youxiang Diao, Yi Tang

**Affiliations:** ^1^College of Animal Science and Technology, Shandong Agricultural University, 61 Daizong Street, Tai'an, Shandong Province 271018, China; ^2^Shandong Provincial Key Laboratory of Animal Biotechnology and Disease Control and Prevention, Tai'an, Shandong 271018, China; ^3^Shandong Provincial Engineering Technology Research Center of Animal Disease Control and Prevention, Tai'an, Shandong 271018, China

## Abstract

Novel duck orthoreovirus can infect various hosts, mainly causing spleen necrosis and hemorrhagic necrotizing hepatitis, leading to immunosuppression and growth impairment. Infectious diseases characterized by swollen hock joints, movement disorders, and impaired growths have recently occurred in significant duck breeding areas in China. Infected ducks from Shandong Province were selected, and N-DRV-LY20 was isolated from the swollen joints. Next-generation sequencing (NGS) of N-DRV-LY20 and homology analysis showed that all ten fragments had the highest homology to N-DRV and the closest affinity to Duck/N-DRV-XT18/China/2018. After artificial inoculation with N-DRV-LY20 virus solution, Cherry Valley ducks showed the same symptoms of leg swelling and hock joint deformation, which were not explored in previous studies. A long history of evolution has led to significant variation in DRV and altered its pathogenicity. In this study, we confirmed the ability of N-DRV to cause viral arthritis in ducks and clarified this isolate's genetic characteristics and level of variation, providing a basis for the control and variation detection of N-DRV.

## 1. Introduction

Avian orthoreovirus (ARV) was first successfully isolated in China in 1991 [1]. ARV belongs to the family *Eutheroviridae*, subfamily *Spiny Eutherovirus*, and genus *Orthoreovirus* [2], which has no envelope, and the viral particles are icosahedral symmetric sphere [3]. The virus induces cell fusion [4, 5], lacks hemagglutination activity [6], and leads to immunosuppression in poultry, secondary to other pathogenic infections [7]. A variety of avian species can be infected with ARV, such as chickens, turkeys, ducks, geese, pigeons, and wild birds [7–10], causing viral arthritis, tenosynovitis, growth retardation, and chronic respiratory diseases [11]. Based on sequence nucleotide homology and evolutionary analysis, waterfowl orthoreovirus (WRV) can be classified into two genotypes. Muscovy duck orthoreovirus (MDRV) and goose orthoreovirus (GRV) are classified as waterfowl orthoreovirus genotype 1 [12, 13]. Novel goose orthoreovirus (N-GRV) and novel duck orthoreovirus (N-DRV) are classified as waterfowl orthoreovirus genotype 2 [14].

Novel duck orthoreovirus is an infectious disease that mainly causes spleen necrosis in ducks, which is prevalent mainly in Hebei, Shandong, Jiangsu, Anhui, Zhejiang, Inner Mongolia, and Fujian [15, 16]. N-DRV has a wide range of natural hosts, can be transmitted horizontally or vertically, and has a high morbidity and mortality rate in ducklings. N-DRV has the same cultural characteristics as ARV that can be isolated and proliferated in various ways [17]. There are ten segments of double-stranded RNA in the genome of N-DRV, which are divided into three groups: large segments (L1, L2, and L3), medium segments (M1, M2, and M3), and small segments (S1, S2, S3, and S4) [18, 19]. The L fragment encodes *λ* proteins (*λ*A, *λ*B, and *λ*C), the M fragment encodes *μ* proteins (*μ*A, *μ*B, and *μ*NS), the S fragment encodes *σ* proteins (*σ*C, *σ*A, *σ*B, and *σ*NS), and the S1 gene additionally encodes proteins *p*10 and *p*18 [4, 20–22]. Current studies of the N-DRV genome sequence have focused on the S-segment. The S1 gene encodes the *σ*C protein, an adhesion protein located on the surface of the viral capsid [4], which attaches to host cells and is a significant site for binding cellular receptors, and also induces apoptosis [23]. In addition, the *σ*C protein induces neutralizing antibodies against orthoreovirus [24]. The main functions of each protein encoded by each gene segment are shown in Table S1.

Viral arthritis in ducks has been emerging in the duck breeding areas of east-central China for the past two years, with the scope of infection expanding, characterized by swollen hock joints, movement disorders, decreased immune function, and impaired growth. Dissection revealed severe joint swelling and necrosis of the liver and spleen. To determine the pathogen causing the disease, we separately isolated viruses and bacteria from the swollen joints of diseased ducks; and finally, obtained the stably surviving virus strain. After whole genome sequencing and evolutionary genetic analysis, we identified the isolated virus as a novel orthoreovirus. The pathogenicity of this isolate was subsequently studied by artificially infecting Cherry Valley meat ducks. It was found that although the N-DRV-LY20 was genetically similar to the Duck/N-DRV-XT18/China/2018, the clinical symptoms caused were significantly different. This study improves our understanding of avian orthoreovirus's evolutionary relationships and diversity. It provides a scientific basis for preventing and controlling new orthoreovirus infections in ducks and lays the foundation for further understanding the pathogenic mechanisms of orthoreovirus.

## 2. Materials and Methods

### 2.1. Ethics Statement

All the animal infection experiments were approved by the Animal Care and Use Committee of Shandong Agricultural University and conducted following the “Guidelines for Experimental Animals” of the Ministry of Science and Technology (Beijing, China).

### 2.2. Clinical Samples

Tendon samples of diseased ducks characterized by arthritis were used for virus isolation in Shandong Province, China. The existence of common avian pathogenic viruses such as avian influenza virus, duck virus hepatitis A types 1, duck virus hepatitis A types 3, Newcastle disease virus, avian orthoreovirus, and duck plague virus was excluded by the RT-PCR or PCR methods (Table S2) [25].

### 2.3. RNA Extraction and RT-PCR

According to the manufacturer's instructions, total RNA from the tissue filtrate was extracted using the RNAprep Pure Tissue Kit (TIANGEN, Beijing, China). Extracted RNA samples were amplified using One-Step RT-PCR Kit (TaKaRa, Dalian, China) with specific primers (N-DRV-F 5′-ATGGATCGCAACGAGGTGATAC-3′, N-DRV-R 5′-CTAGCCCGTGGCGACGGT-3′) to a partial sequence of 966 bp in the N-DRV *σ*C gene with the following reaction conditions: 42°C for 50 min, 94°C for 5 min, 94°C for 30 s, 54°C for 30 s, 72°C for 45 s, and 32 cycles. Beijing Genomics Institution (BGI) synthesized all primers. The PCR products were collected by electrophoresis in 1.0% agarose gel, and PCR-amplified fragments were sequenced and confirmed by Sanger sequencing (TsingKe Biotech, Beijing, China) for confirmation.

### 2.4. Viral Isolation

The collected tendon and swollen joint samples were processed by grinding and homogenizing after adding four times saline. The supernatant solution obtained after centrifugation was repeatedly frozen and thawed three times. We used LMH (Leghorn Male-chicken Hepatocellular-carcinoma, ATCC CRL-2117) cells inoculated with treated supernatant, Dulbecco's Modified Eagle Medium (DMEM; Hyclone, Beijing, China) containing 2% fetal bovine serum (FBS; Hyclone, Beijing, China) was used as a cell growth maintenance solution, and the culture conditions were 37°C and 5% CO_2_. Virus cultures were collected and continuously re-cultured until stable CPE was observed. After sufficient dilution of the virus solution, the virus was purified using the plaque purification technology, and the final purified cell culture was frozen.

The titer of N-DRV-LY20 in LMH cells was determined according to the half-tissue culture infectious dose (TCID_50_). LMH cells were seeded in 96-well plates, inoculated with 10^5^ cells per well, and incubated for 48 h at 37°C under 5% CO_2_. The viral solution was serially diluted 10-fold, and 100 *μ*l was added to each well. Measure CPE 4 days after inoculation. Virus titers were determined according to the method of Reed and Muench [26].

### 2.5. Next-Generation Sequencing and Analysis

Infected cell cultures of the N-DRV-LY20 strain were extracted from viral RNA using the TruSeq Stranded Total RNA Sample Prep Kit (Illumina, Shanghai, China) to construct sequenced RNA libraries. The library fragments were purified using the Ampre XP (Beckman-Coulter) and quantified using the Bioanalyzer 2100 system [7, 27]. The prepared cDNA libraries were loaded onto the Illumina MiSeq platform to assemble the viral genome, reads of nontarget genes were removed, and the raw reads of NGS data were analyzed. Assemble all pure reads using QIAGEN software and align sequences. Virus (ORF) prediction, amino acid (aa) translation, and nucleotide (nt) sequence alignment were performed using Editseq software in DNASTAR software and Megalign software [28, 29]. The BLAST program on the NCBI website was used to search for published related isolates and further analyze homology relationships. Information on the viral strains used in the analysis is presented in Table S3. Genetic evolution trees were constructed using the Clustal W program in MEGA 11 software.

### 2.6. Animals and Samples Collection

One hundred and twenty healthy Cherry Valley ducklings (1 day old) were purchased from Liuhe Jingwei Agricultural and Livestock Company in Tai'an, Shandong Province. The ducklings were tested negative for N-DRV and other pathogens in blood and cloacal swabs using qPCR and negative for antibodies to N-DRV in serum using ELISA before the experiment. They were randomly divided into four groups of thirty ducks each. Three experimental groups (A, B, and C) were set up to be inoculated with the virus by footpad, intramuscular, and oral administration. Each duckling was inoculated with 0.2 ml of N-DRV-LY20 virus (TCID_50_: 10^−5.8^/0.1 ml), and Group D was inoculated with 0.2 ml of saline as the control group. Later, all ducklings were continuously observed daily and recorded clinical symptoms, weight changes, and mortality following infection.

Three ducks in each group were euthanized by CO_2_ on days 1, 3, 5, 7, 10, 14, 18, and 22 following infection, and blood and cloacal swabs were collected. Various tissue specimens were collected from Fabricius, the thymus, and the tendons' liver, spleen, lung, kidney, and bursa. One part of the tissue sample set was also fixed in 10% neutral buffered formalin to make paraffin sections. Sections were examined microscopically with hematoxylin and eosin staining.

### 2.7. Genomic RNA Extraction and Detection of Viral Load

The extraction of total RNA from all samples was performed using the MiniBEST Universal RNA Extraction Kit (TIANGEN, Beijing, China) according to the instructions, and the concentrations were measured using a DeNovix DS-11 spectrophotometer (Nanodrop, USA) before being stored at −80°C. The viral load was detected using the N-DRV TaqMan probe fluorescent quantitative RT-PCR method established previously in our laboratory [25, 30]. The primers and the TaqMan probe (Table S2) used to detect the viral load were designed based on the S1 gene of the N-DRV strain.

One-Step PrimeScript™ RT-PCR kit (TaKaRa, Dalian, China) was used to perform qPCR reactions. The reaction system contained 2 × One-Step RT-PCR Buffer III 10 *μ*l, 0.4 *μ*l each of TaKaRa Ex Taq HS, PrimeScript RT Enzyme Mix II, PCR Forward primer and PCR Reverse primer, total RNA 80 ng. Finally, RNase-free H_2_O was added to make a total volume of 25 *μ*l. Roche LightCycler 96 Real-Time PCR System (Roche, Switzerland) system was used with a reaction program of 42°C for 5 min, 95°C for 10 s, 95°C for 5 s, and 60°C for 20 s for 40 cycles, and fluorescence signals were collected.

### 2.8. Antibody Level Detection

We used an indirect ELISA method to detect N-DRV antibody levels. The blood was first incubated at 37°C for 1 h and centrifuged at 3000 r/min for 10 min to collect the serum. The 96-well plate was precoated with 2000-fold dilution N-DRV-*σ*C protein in 0.05 M carbonate buffer (*pH* 9.6), then washed three times with PBST (0.01 M PBS, *pH* 7.2, 0.05% tween 20) for 3 min each time the next day. Add 200 *μ*l blocking solution (5% skim milk powder) and leave at 37°C for 1 h. Dilute serum 1 : 20 times with 5% skim milk powder, add 100 *μ*l per well and leave at 37°C for 1 h. Dilute HRP-labeled antiduck secondary antibody (KPL) (Solarbio, China) 1 : 500 times with 5% skim milk powder, then add 100 *μ*l per well and leave at 37°C for 1 h. Add 100 *μ*l of TMB color development solution under light-proof conditions and let it stand for 15 min at 37°C before adding 50 *μ*l of termination solution (2 M sulfuric acid). The final reading was taken at an OD value of 450 using a microplate reader.

### 2.9. Statistical Analysis

Data on body weight, viral load, and blood antibody levels were obtained and statistically analyzed using GraphPad Prism software 9.0 (GraphPad Software Inc.) and were expressed as means ± SD. The data from all experimental and control groups were compared in the same graph. *P* < 0.05 was considered a statistically significant difference.

## 3. Results

### 3.1. N-DRV Isolation and Identification

**Figure 1:**
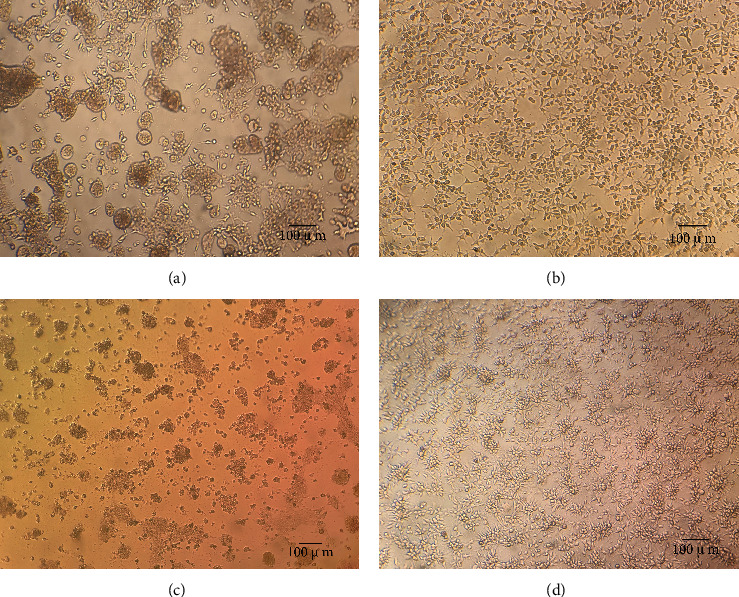
CPE of LMH cells infected with the N-DRV-LY20. (a) 24 h infected diseased cells; (b) 24 h control cells; (c) 72 h infected diseased cells; (d) 72 h control cells.

Third-generation cell cultures showed obvious cytopathic effects (CPE), with clear syncytial features at 24 h, and 90% of cells showed cell shedding at about 72 h (Figure 1). ARV causes rounding and shedding of LMH cells, forming a characteristic giant or bloom-like subrounded syncytial CPE [31]. Using RT-PCR amplification (with F/R primers based on the S1 gene design), we confirmed that the cell culture tested N-DRV positive. We named the isolate Duck/N-DRV-LY20/China/2021. Meanwhile, RT-PCR or PCR was used to detect other common pathogens, and the results showed that all other viruses tested negative. According to the method of Reed and Muench, the virus titer was determined as TCID_50_ = 10^−5.8^/0.1 ml.

### 3.2. NGS Analysis

After viral genome assembly, we obtained 10 contigs that covered the full length of ARV. The entire genome of the N-DRV-LY20 strain was 23,419 bp in length and contained 10 genomic fragments ranging in size from 1191 bp (S4) to 3959 bp (L1). Ten segments of the whole genome sequence of the N-DRV-LY20 strain were uploaded to GenBank with the accession numbers ON040907 (L1) to ON040916 (S4). We determined ORFs by predicting each segment's ORF start and stop codons. The S1 gene contained three ORFs for the *p*10 (98aa), *P*18 (163aa), and *σ*C (322aa) proteins, and the remaining gene segments contained only one ORF, each encoding one protein (Table 1). Sequence analysis of the 5′ and 3′ untranslated regions (UTR) showed that the nucleotide sequences of the fragments of the N-DRV-LY20 isolate remained conserved. They were consistent with the published sequences for ARV, DRV, and GRV: the identical GCUUUUU sequence at the 5′ UTR and the UCAUC sequence at the 3′ UTR. No other pathogens were found during the analysis of the samples.

### 3.3. Phylogenetic Analysis

Nucleotide sequences of the N-DRV-LY20 strain were analyzed for nucleotide (nt) and amino acid (aa) homology with avian-derived, goose-derived, and duck-derived orthoreoviruses published on GenBank (Table S4). The results showed that strain LY20 had the highest homology with N-DRV among all fragments and was most closely related to strain N-DRV-XT18. The S1 gene had the lowest homology with other sequences, a relatively small genetic distance among strains, and a close genetic evolutionary relationship with the ARVbranch because the S1 gene encodes the *σ*C protein of ARV and DRV, whereas the *σ*C protein of MDRV is encoded by the S4 gene [4]. The homology comparison showed that the S1 gene of the N-DRV-LY20 had higher homology with the N-DRV-XT18 (nt: 98.3%; aa: 97.7%) and lower homology with the ARV/S1133 (nt: 45.2%; aa: 11.3%) and the MDRV/815-12 (nt: 33.4%; aa: 14.5%). Similarly, the *σ*C gene of N-DRV-LY20 had the highest homology with the N-DRV-XT18 (nt: 98.9%; aa: 99.1%) and lower homology with the MDRV/815-12 (nt: 49.6%; aa: 42.2%) and the ARV/S1133 (nt: 39.6%; aa: 28.3%).

**Figure 2:**
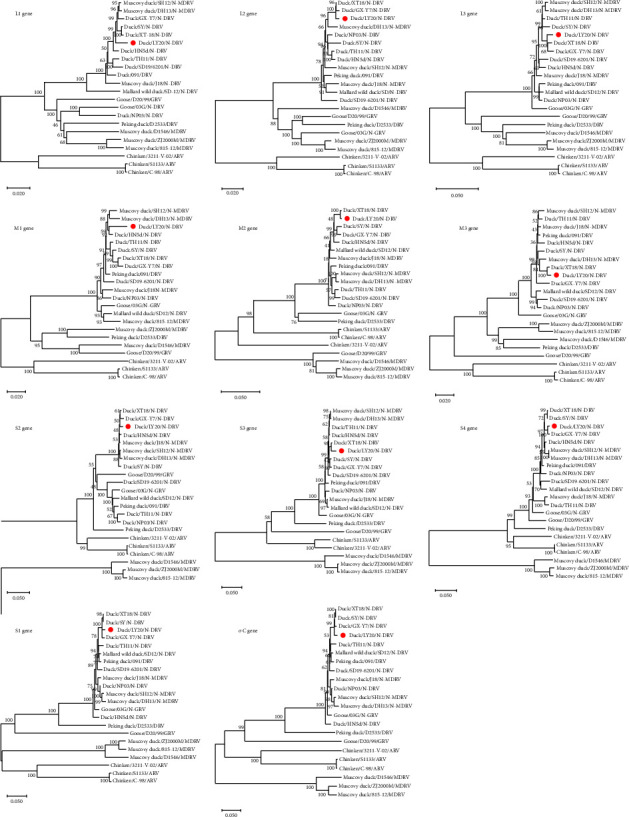
Phylogenetic trees of the N-DRV-LY20. Phylogenetic trees are constructed based on orthoreovirus species' nucleotide sequences of the homologous gene segments. N-DRV-LY20 isolate was labeled using a red circular pattern.

The results of the genetic evolution analysis showed that the avian orthoreovirus formed three evolutionary branches, namely, the ARV branch, MDRV branch, and N-DRV branch, in the genetic evolution analysis of 10 segments (Figure 2). In the evolutionary genetic analysis tree of L1, L2, L3, M1, M3, S2, and S4 genes, N-DRV was more closely related to MDRV, and ARV was in a separate branch. In the evolutionary genetic analysis tree of the M2, S1, and S3 genes, N-DRV was more closely related to ARV, and MDRV was in a separate evolutionary branch.

### 3.4. Pathogenicity of N-DRV-LY20 in Ducklings

**Figure 3:**
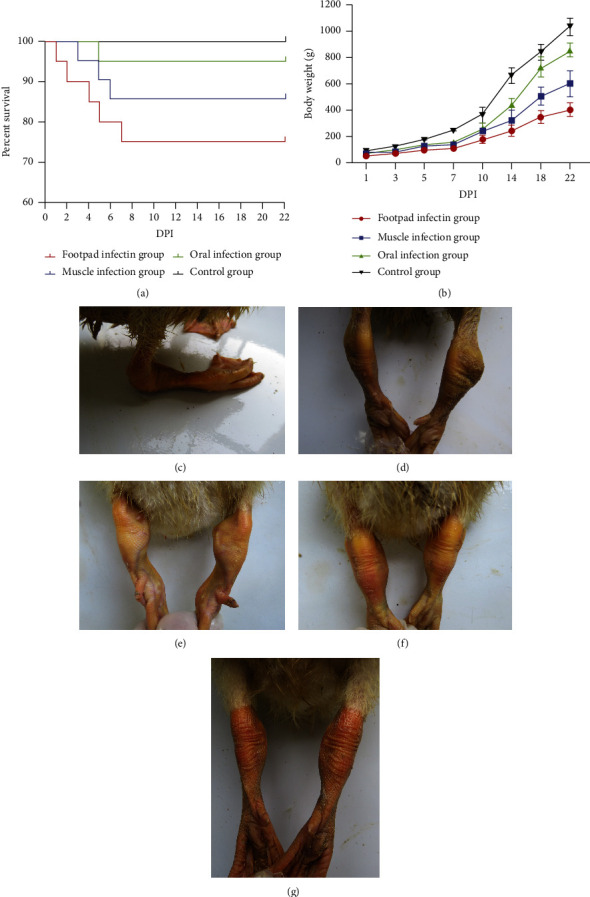
Clinical symptoms, weight changes, and mortality of infected ducklings. (a) Survival curves; (b) the footpad injection group had the most severe inhibitory effect on body weight, and the oral administration group had slightly lower body weight than the control group; (c) sick duck paralyzed and unable to stand; (d) severe swelling of the joints; (e) severe deformation of the joints; (f) thickening of joints; (g) normal joints.

Thirty days of clinical sign monitoring were used to study the pathogenicity of the N-DRV-LY20 isolate in Cherry Valley ducklings. After ducklings were infected with N-DRV, the clinical signs caused by different inoculation methods were similar, with the most severe signs in the footpad injection group. The morbidity rate in the infected group was 100%, and mortality rates differed, with 30% in the footpad injection group, 20% in the intramuscular injection group, and 17% in the oral group. All deaths of ducklings were concentrated within 7 dpi (Figure 3(a)). The weight monitoring results indicated that artificially infected ducklings' weight gain was suppressed (Figure 3(b)). The symptoms were mainly mental lethargy, decreased feeding and drinking, loose white stools, slow growth, and a sparse coat of the affected ducks. In addition, diseased ducks showed severe movement disorders with obvious deformation of joints or paralysis (Figures 3(c)–3(f)).

**Figure 4:**
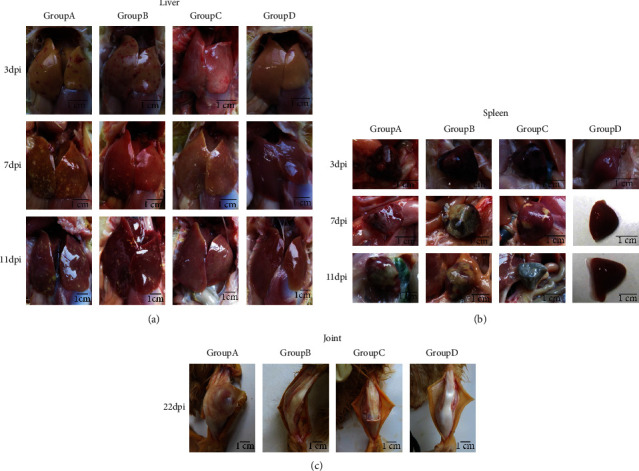
Changes in the dissection of different tissues and organs. (a) Characteristics of the liver in different periods and groups; (b) spleen characteristics of different periods and groups; (c) anatomical condition of the joints of the sick duck, with severe deformation and swelling.

It was observed that the symptoms caused by different inoculation methods were consistent with the naturally infected ducks. Liver necrosis existed in all groups of ducklings (Figure 4(a)). Hemorrhagic diffuse necrotic spots of variable size with black necrotic foci visible at the margins were seen on the liver. The spleen was severely lesioned (Figure 4(b)), swollen at 1 dpi, appeared black necrotic spots at 2 dpi, and had lost its original texture and color, turning gray-black at 3 dpi dissection of the joints showed subcutaneous hemorrhage, suppuration of the joint cavity, and tibial deformation (Figure 4(c)). Other tissues and organs were damaged to different degrees, mainly showing intestinal hemorrhage and thinning of the intestinal wall; bleeding and black necrotic spots in the lungs; swollen and hemorrhage in the thymus; and a swollen hemorrhagic kidney.

### 3.5. Pathological Changes of N-DRV-LY20 in Ducklings

**Figure 5:**
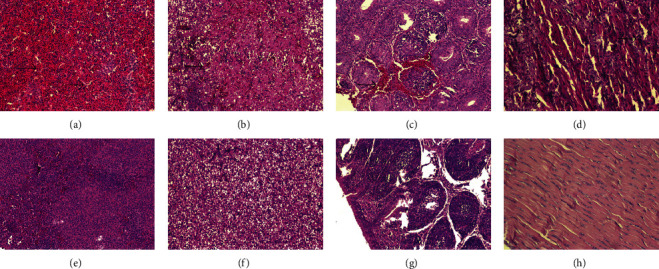
Histopathological changes in different organs. Routine HE staining and histopathological changes were observed under light microscopy. Magnification is 100x. (a) Massive erythrocyte sludge in the spleen tissue, necrosis, and necrotic foci was mechanized; (b) swelling of liver cells, necrosis, and destruction of parenchymal structure; (c) large red blood cells in the bursa of Fabricius tissue; (d) widening and rupture of tendon fibers, structural destruction of tendon fibers; (e–h) corresponding tissue control.

Different virus inoculation methods caused no significant difference in pathological changes; no abnormal changes were seen in the control group. The spleen was extensively necrotic and subsequently replaced by fibrous tissue. The demarcation between the cortex and medulla was blurred, with massive erythrocyte filling, increased macrophages, and inflammatory cell infiltration (Figure 5(a)). It showed steatosis, cellular hypertrophy or atrophy, disorganized hepatic cords, hepatic sinus hemorrhage, neutrophil infiltration, lymphocytosis, and foci of coagulative necrosis in the liver (Figure 5(b)). The bursa of Fasciola showed interstitial edema with reduced lymphocytes, and scattered inflammatory cells in the necrotic area (Figure 5(c)). Tendon fibers were widened, broken, and lost their original fibrous structure. Inflammatory cells proliferated in the tendon sheath (Figure 5(d)). Widening and degeneration of interstitial myocardial fibers with inflammatory cell infiltration. The renal tubules and the glomeruli are necrotic and atrophic, with neutrophil infiltration, epithelial swelling of the renal tubules, narrowing of the tubular lumen, and interstitial vasodilatation or congestion. The thymus was hemorrhagic with erythrocyte sludge. The alveolar tissue is destroyed or atrophied, and lymphocytes predominantly infiltrate the inflammatory cells.

### 3.6. Viral Replication and Shedding Pattern

**Figure 6:**
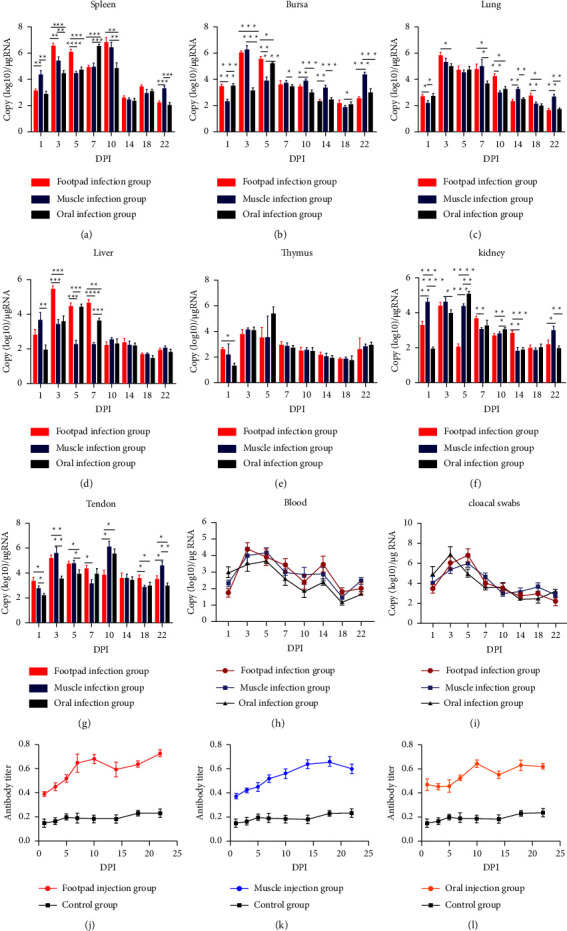
Tissue viral content and antibody levels. The data patterns for the three different inoculation methods are indicated using different colors. (a–g) Determination of virus content in the spleen, bursa of Fabricius, the lung, tendon, liver, thymus, and kidney; (h, i) determination of virus content in blood samples and cloacal swab samples; (j–l) results of the ELISA test for N-DRV antibody levels in serum. Data are expressed as mean ± SEM. All experiments were repeated at least three times, and the results were similar. ^*∗*^*P* < 0.05, ^*∗∗*^*P* < 0.01, ^*∗∗∗*^*P* < 0.001, ^*∗∗∗∗*^*P* < 0.0001, indicating a statistically significant difference compared between the different groups.

The viral replication pattern in various tissues and the shedding pattern of Cherry Valley ducks infected with the N-DRV-LY20 can be derived using TaqMan probe qPCR at different times and organs. Virus levels and their changes were more consistent with different inoculation methods but not similar between different organs (Figures 6(a)–6(g)). Viral content in the spleen was significantly higher than in other organs. Positive data could be monitored as early as 1 dpi in all organs and continued to rise for several days before reaching a peak, followed by a gradual decrease, and then maintained at a relatively stable level. The spleen, lung, and liver maintained high viral levels at 3–7 dpi. Differently, the bursa of Fabricius, the thymus, and kidney had elevated levels at 3–5 dpi, while a small peak occurred at 22 dpi in the bursa of Fabricius and the thymus. When comparing the three infection groups, it was found that the virus replication rate of the organs in the oral group rose slower than in the other two groups.

It is rapid for the virus to enter the bloodstream and excrete from the body, with similar trends in virus levels and changes between various infection groups. High amounts of the virus could be detected in blood and cloacal swab samples at 1 dpi (Figures 6(h)–6(i)). Virus levels in the blood increased rapidly over 3 dpi, and then slowly decreased until a rebound occurred at 14 dpi. There was also a tendency for the virus number in the cloaca to rising then fall. The peak in groups A and B occurred at 5 dpi, while group C occurred at 3 dpi, and the virus content then gradually decreased but still showed two slight rises at 14 dpi and 22 dpi.

### 3.7. Antibody Response in Affected Ducks

The ELISA method measured the antibody levels in the serum of infected ducks to understand the protective effect produced against the virus. All infected groups differed from the control group regarding antibody levels and response effects. A comparison of the different experimental groups showed that the antibody levels changed fastest in the footpad injection group, while they were produced least in the oral group, but still higher than the control group. The changes in antibody levels in the footpad injection group were similar to those in the control group, with the highest antibody levels at 10 dpi (Figure 6(j)). For the intramuscular group, antibody levels tended to increase after inoculation, reaching a maximum of 18 dpi, where the changes were more moderate (Figure 6(k)). While for the oral group, there is a distinct peak at 10 dpi and two less distinct peaks at 1 dpi and 18 dpi (Figure 6(l)).

## 4. Discussion

Avian orthoreovirus (ARV) was first isolated in 1954 and reported in China in 1985, which frequently causes viral arthritis and tenosynovitis in various avian species [32]. Several waterfowl orthoreovirus have been isolated successively in China, showing significant differences in host and pathogenicity, separately causing white spot disease, hemorrhagic necrotizing hepatitis, and spleen necrosis [13, 14]. The novel duck orthoreovirus was first reported to cause duck spleen necrosis in 2016 [33]. Over the past decade, the disease has become a severe disease that seriously affects the duck breeding industry, evolving with increasing lethality and different clinical features [25, 34]. Diseased ducks rapidly contaminate feed and drinking water through the digestive and respiratory tracts. Tolerance of the virus to the environment allows it to survive for a long time, resulting in a wider spread of the pathogen. In the past two years, we have found a frequent occurrence of viral arthritis in major duck areas in China, mainly characterized by swollen joints, unstable standing, and impaired growth. We finally confirmed that it was caused by novel duck orthoreovirus through pathogen isolation and identification, which differed from the previously reported symptoms. The connection between the novel duck orthoreovirus and viral arthritis was not discussed previously.

After successfully isolating Duck/N-DRV-LY20/China/2021, we sequenced the entire genome using NGS and compared it with other orthoreovirus species at the nucleotide and amino acid levels. Data analysis showed that the 10 genomic fragments of N-DRV-LY20 were the same length as the homologous fragments of the previous N-DRV isolates, with highly conserved sequences at the 5′ and 3′ ends, and showed the closest affinity to N-DRV-XT18. The genetic relationship of the L segment is stable. Still, the significant amino acid variation suggests that most of the nucleotides of the L gene should be synonymous mutations, excluding the variable region. The *σ*C protein has a significant role in viral attachment and becomes the most variable part of all ARV proteins under frequent selection pressure [23]. However, the variation was not significant in N-DRV-LY20 compared with N-DRV-XT18 between *σ*C gene fragments (nt: 98.9%; aa: 99.1%) but more prominent between M1 fragments (nt: 96.1%; aa: 90.7%), suggesting that a new highly variable region may exist in M1 gene fragments.

To better understand the pathogenicity of N-DRV-LY20, we successfully conducted animal regression experiments in Cherry Valley ducks, and the virus was inoculated by footpad, intramuscular, and oral injection routes. All groups consistent with the natural disease state showed severe swelling of the leg joints and marked necrosis of the liver and spleen. Ducks inoculated via footpad were sometimes more susceptible to N-DRV than intramuscularly, especially when the duck's flippers were ruptured and infected by the contaminated surroundings [35]. The footpad injection group caused the most severe symptoms, showing significant mobility impairment, leg swelling, joint abscesses and ulcers at 10 dpi, and paralysis at 18 dpi. The N-DRV-LY20 could also cause splenic necrosis disease rapidly like the N-DRV-XT18, and severe liver necrotic spots similar to the NP03 strain [15, 34, 36]. Furthermore, diseased ducks exhibit other immune organ damage, such as a swollen bursa of Fabricius and thymic hemorrhage. Wang recently reported that the N-DRV-XT18 strain has extensive tissue orientation and may cause severe damage to immune organs [37, 38]. In addition, N-DRV can cause decreased immune function and is more likely to cause secondary infections with other pathogens [38].

There was 100% morbidity in the infected group, but mortality and weight change were significantly influenced by the route of vaccination and age at infection. The highest mortality was in the footpad injection group (30%), followed by the intramuscular injection group (20%), and the oral group (17%), which all appeared within 7 dpi different DRV strains isolated in China in recent years differed widely in the mortality rates, with rates of 10%–15% in Pekin ducks earlier discovered, rising to 40% in 2011 for the TH11 strain and up to 60% in 2013 for the variant HN5d strain [39]. The pattern of pathogenicity was consistent, but the mortality was higher compared to the N-DRV-XT18. Body weight changes indicate that ducklings infected with the N-DRV virus by any route showed weight gain suppression and poor growth, especially in the footpad-inoculated group.

The virus was detected in all tissues after inoculating the viral solution, with higher viral loads in the spleen and bursa of Fabricius, suggesting that the spleen and bursa may be our organs of choice for clinical testing of N-DRV. N-DRV destroys the spleen and bursa of Fasciola, reduces immune function, accelerates the invasion of other pathogens, and causes secondary infections. More minor ages of infection cause more severe disease reactions, and when over two weeks, it tends to be more easily tolerated and then becomes a stiff duck. Infected duck excrement contaminates feed and drinking water, which can easily cause mass transmission in the duck flock. When the disease is found in production, disinfection measures should be done, and disinfection efforts should be increased to prevent the spread of pathogens.

Overall, the N-DRV-LY20 isolate was able to cause duck viral arthritis with altered pathogenicity in Cherry Valley ducks compared to previously reported strains. Long-term evolution has caused significant variation in DRV. Detailed exploration of the genome and pathogenicity of the N-DRV-LY20 isolate provides a basis for further investigation of the pathogenesis of the newly identified N-DRV variant. To control the continued occurrence and prevalence of N-DRV, persistent monitoring of the genes and pathogenicity of the virus is needed, as well as efficient vaccines for prevention and treatment. Further studies on the mechanisms of enhanced virulence and genome reorganization of the duck orthoreovirus are also needed.

## Figures and Tables

**Table 1:** The complete genomic sequence of N-DRV/LY20.

Genome segment	Length (bp)	ORFc (nt)	Coding protein	Protein length (aa)
L1	3959	22–3903	*λ*A	1293
L2	3830	15–3794	*λ*B	1259
L3	3907	13–3870	*λ*C	1285
M1	2284	14–2212	*μ*A	732
M2	2158	30–2057	*μ*B	675
M3	1996	25–1932	*μ*NS	635
S1	1568	20–313	*P*10	97
		273–761	*P*18	162
		571–1536	*σ*C	321
S2	1324	16–1266	*σ*A	416
S3	1202	31–1134	*σ*B	367
S4	1191	24–1127	*σ*NS	367
Total	23419			

## Data Availability

All data in this study are available on request.

## Supplementary Materials

Supplementary Materials Supplementary Table S1. Main functions of proteins encoded by orthoreovirus genes. Supplementary Table S2. Primers for PCR in this study. Supplementary Table S3. GenBank accession numbers of avian orthoreoviruses for sequence alignment. Supplementary Table S4. Homology analysis between LY20 strain and other strain (%).

## References

[1] Li-Kuan C., Xin C., Huang W. M. (1991). Isolation and identification of chicken viral arthritis virus. *Chinese Journal of Virology*.

[2] King A. M. Q., Lefkowitz E., Adams M. J., Carstens E. B. (2011). *Virus Taxonomy: Ninth Report of the International Committee on Taxonomy of Viruses*.

[3] Spandidos D. A., Graham A. F. (1976). Physical and chemical characterization of an avian reovirus. *Journal of Virology*.

[4] Bodelón G., Labrada L., Martínez-Costas J., Benavente J. (2001). The avian reovirus genome segment S1 is a functionally tricistronic gene that expresses one structural and two nonstructural proteins in infected cells. *Virology*.

[5] Duncan R., Sullivan K. (1998). Characterization of two avian reoviruses that exhibit strain-specific quantitative differences in their syncytium-inducing and pathogenic capabilities. *Virology*.

[6] Gillian A. L., Nibert M. L. (1998). Amino terminus of reovirus nonstructural protein *ς*NS is important for ssRNA binding and nucleoprotein complex formation. *Virology*.

[7] Tang Y., Lu H. (2015). Genomic characterization of a novel avian arthritis orthoreovirus variant by next-generation sequencing. *Archives of Virology*.

[8] Simmons D. G., Colwell W. M., Muse K. E., Brewer C. E. (1972). Isolation and characterization of an enteric reovirus causing high mortality in Turkey poults. *Avian Diseases*.

[9] Vindevogel H., Meulemans G., Pastoret P. P., Schwers A., Calberg-Bacq C. M. (1982). Reovirus infection in the pigeon. *Annales De Recherches Veterinaires Annals of Veterinary Research*.

[10] Zhu Y. Q., Li C. F., Bi Z. L. (2015). Molecular characterization of a novel reovirus isolated from Pekin ducklings in China. *Archives of Virology*.

[11] Neelima S., Ram G. C., Kataria J. M., Goswami T. K. (2003). Avian reovirus induces an inhibitory effect on lymphoproliferation in chickens. *Veterinary Research Communications*.

[12] Palya V., Glávits R., Dobos-Kovács M. (2003). Reovirus identified as cause of disease in young geese. *Avian Pathology*.

[13] Wang D., Xu F., Ma G. (2012). Complete genomic sequence of a new muscovy duck-origin reovirus from China. *Journal of Virology*.

[14] Wang D., Shi J., Yuan Y., Zheng L., Zhang D. (2013a). Complete sequence of a reovirus associated with necrotic focus formation in the liver and spleen of Muscovy ducklings. *Veterinary Microbiology*.

[15] Liu Q., Zhang G., Huang Y. (2011). Isolation and characterization of a reovirus causing spleen necrosis in Pekin ducklings. *Veterinary Microbiology*.

[16] Li N., Hong T., Wang Y. (2016). The pathogenicity of novel duck reovirus in Cherry Valley ducks. *Veterinary Microbiology*.

[17] Wood G. W., Nicholas R., Hebert C. N., Thornton D. H. (1980). Serological comparisons of avian reoviruses. *Journal of Comparative Pathology*.

[18] Kuntz-Simon G., Le Gall-Reculé G., de Boisséson C., Jestin V. (2002). Muscovy duck reovirus *σ*C protein is atypically encoded by the smallest genome segment. *Journal of General Virology*.

[19] Ma G., Wang D., Shi J., Jiang T., Yuan Y., Zhang D. (2012). Complete genomic sequence of a reovirus isolate from Pekin ducklings in China. *Journal of Virology*.

[20] Benavente J., Martínez-Costas J. (2007). Avian reovirus: structure and biology. *Virus Research*.

[21] Schnitzer T. J., Ramos T., Gouvea V. (1982). Avian reovirus polypeptides: analysis of intracellular virus-specified products, virions, top component, and cores. *Journal of Virology*.

[22] Varela R., Martínez-Costas J., Mallo M., Benavente J. (1996). Intracellular posttranslational modifications of S1133 avian reovirus proteins. *Journal of Virology*.

[23] Shih W. L., Hsu H. W., Liao M. H., Lee L. H., Liu H. J. (2004). Avian reovirus *σ*C protein induces apoptosis in cultured cells. *Virology*.

[24] Labrada L., Bodelón G., Vin˜uela J., Benavente J. (2002). Avian reoviruses cause apoptosis in cultured cells: viral uncoating, but not viral gene expression, is required for apoptosis induction. *Journal of Virology*.

[25] Wang H., Gao B., Chen H., Diao Y., Tang Y. (2019). Isolation and characterization of a variant duck orthoreovirus causing spleen necrosis in Peking ducks, China. *Transbound Emerg Dis*.

[26] Reed L. J., Muench H. (1938). A simple method of estimating fifty per cent endpoints l 2. *American Journal of Epidemiology*.

[27] Wei F., Yang J., Wang Y., Chen H., Diao Y., Tang Y. (2020). Isolation and characterization of a duck-origin goose astrovirus in China. *Emerging Microbes & Infections*.

[28] Tang Y., Lin L., Sebastian A., Lu H. (2016). Detection and characterization of two co-infection variant strains of avian orthoreovirus (ARV) in young layer chickens using next-generation sequencing (NGS). *Scientific Reports*.

[29] Yang J., Tian J., Chen L., Tang Y., Diao Y. (2018). Isolation and genomic characterization of a novel chicken-orign orthoreovirus causing goslings hepatitis. *Veterinary Microbiology*.

[30] Zhang S., Li W., Liu X. (2020). A TaqMan-based real-time PCR assay for specific detection of novel duck reovirus in China. *BMC Veterinary Research*.

[31] Sterner F. J., Rosenberger J. K., Margolin A., Ruff M. D. (1989). In vitro and in vivo characterization of avian reoviruses. II. Clinical evaluation of chickens infected with two avian reovirus pathotypes. *Avian Diseases*.

[32] Fahey J. E., Crawley J. F. (1954). Studies on chronic respiratory disease of chickens II. Isolation of A Virus. *Canadian Journal of Comparative Medicine and Veterinary Science*.

[33] Zhao J. Q., Wan P., Xiang J. (2011). Synthesis of highly ordered macro-mesoporous anatase TiO2 film with high photocatalytic activity. *Microporous and Mesoporous Materials*.

[34] Wang H., Gao B., Liu X., Zhang S., Diao Y., Tang Y. (2020a). Pathogenicity of a variant duck orthoreovirus strain in Cherry Valley ducklings. *Veterinary Microbiology*.

[35] Chen S. Y., Chen S. L., Lin F. Q. (2012). [The isolation and identification of novel duck reovirus]. *Chinese Journal of Virology*.

[36] Chen S., Chen S., Lin F. (2009). The primary study of pathogen of duck hemorrhagic-necrotic hepatitis. *Chinese Agricultural Science Bulletin*.

[37] Wang H., Liu X., Zhang S., Diao Y., Tang Y. (2019). Pathogenicity of a variant duck orthoorthoreovirus strain in Cherry Valley Ducklings. *Veterinary Microbiology*.

[38] Wang W., Liang J., Shi M. (2020c). The diagnosis and successful replication of a clinical case of Duck Spleen Necrosis Disease: an experimental co-infection of an emerging unique reovirus and Salmonella Indiana reveals the roles of each of the pathogens. *Veterinary Microbiology*.

[39] Zheng X., Wang D., Ning K. (2016). A duck reovirus variant with a unique deletion in the sigma C gene exhibiting high pathogenicity in Pekin ducklings. *Virus Research*.

