# Supplementary material for: Isolation and Pathogenicity Analysis of a Novel Orthoreovirus Caused the Outbreak of Duck Viral Arthritis in China
Source: Transbound Emerg Dis. 2023 May 10;2023:8179312. doi: 10.1155/2023/8179312 (PMC12016782; doi:10.1155/2023/8179312)
Supplement: Supplementary Materials — Supplementary Table S1. Main functions of proteins encoded by orthoreovirus genes. Supplementary Table S2. Primers for PCR in this study. Supplementary Table S3. GenBank accession numbers of avian orthoreoviruses for sequence alignment. Supplementary Table S4. Homology analysis between LY20 strain and other strain (%). [file 8179312.f1.docx]

**Supplementary Materials for**

**Isolation and pathogenicity analysis of a novel orthoreovirus caused the outbreak of duck viral arthritis in China**

Bing Li ^1,2,3#^, Xiaoning Jiang ^1,2,3#^, Shuai Zhang ^1,2,3^, Qianqian Wang ^1,2,3^, Yitong Cui ^1,2,3^, Qiong Wu ^1,2,3^, Wensha Jia ^1,2,3^, Jie Zhang ^1*^, Youxiang Diao ^1,2,3*^, Yi Tang ^1,2,3*^

^1^ College of Animal Science and Technology, Shandong Agricultural University, 61 Daizong Street, Tai'an, Shandong Province, 271018, China

^2^ Shandong Provincial Key Laboratory of Animal Biotechnology and Disease Control and Prevention, Tai'an, Shandong, 271018, China

^3^ Shandong Provincial Engineering Technology Research Center of Animal Disease Control and Prevention, Tai'an, Shandong, 271018, China

^#^ These authors contributed equally to this work.

^*^ Correspondence: Jie Zhang, Youxiang Diao, and Yi Tang, College of Animal Science and Technology, Shandong Agricultural University, Tai'an, Shandong Province 271018, China

Email: zhuji29@163.com; yxdiao@126.com; tyck288@163.com

This file includes: Table S1, S2, S3 and S4

**Table S1.** Main functions of proteins encoded by orthoreovirus genes.

| Genome | Coding | Protein | Major function |
| --- | --- | --- | --- |
|  | Protein | distribution |  |
| L1 | λA | Kernel protein | Nuclear scaffold protein |
| L2 | λB | Kernel protein | Functional activity of RNA polymerase |
| L3 | λC | Kernel protein | The activity of orthoreovirus guanylate transferase |
| M1 | μA | Kernel protein | Transcriptase |
| M2 | μB | Coat protein | Participating in the virus invasion process |
| M3 | μNS | Nonstructural protein | Forming viral factories and recruiting proteins |
| S1 | σC | Coat protein | Promoting adsorption and producing group-specific neutralizing antibodies |
|  | P10 | Nonstructural protein | Membrane fusion proteins that promote syncytium formation |
|  | P18 | Nonstructural protein | Inducing cellular autophagy and enhancing viral replication |
| S2 | σA | Kernel protein | DsRNA binding activity, associated with anti-interferon effects |
| S3 | σB | Coat protein | One of the group specific neutralizing antigens |
| S4 | σNS | Nonstructural protein | Involved in early viral particle assembly |

**Table S2.** Primers used for PCR amplification in this study.

| Name | Sequence of primers(5′to3′) | Length |
| --- | --- | --- |
| N-DRV-F | ATGGATCGCAACGAGGTGATAC | 966 bp |
| N-DRV-R | CTAGCCCGTGGCGACGGT |  |
| AIV-F | GCCATCCTAGCAACGACTGT | 1275 bp |
| AIV-R | CCTGAAGTGCCACAAAATACAA |  |
| DHV-1-F | GGCATGTTGTCAATCGACTCA | 1018 bp |
| DHV-1-R | GTCTCAACCTGATGAACCATTGT |  |
| DHV-3-F | TGATGCGAGTTGGTAA | 800 bp |
| DHV-3-R | CCAACAACCATAATAG |  |
| NDV-F | AGGGACTGAAGAGGAGGATT | 427 bp |
| NDV-R | TGAGTGTGATTGTATTAGGTGG |  |
| GPV-F | GAGCATCAACTCCCGTATGTCC | 640 bp |
| GPV-R | CTACTTCCTGCTCGTCCGTGA |  |
| ARV-F | AGTATTTGTGAGTACGATTG | 1089 bp |
| ARV-R | GGCGCCACACCTTAGGT |  |
| N-DRV-F | CCCGGATTCTCGATGAATGGT |  |
| N-DRV-Probe | FAM-AACGCCTGTGCACGAGCTGAAC-TAMRA | 85 bp |
| N-DRV-R | CGACCCACTGCTGGATACAAG |  |

**Table S3.** GenBank accession numbers of Avian ortho-reoviruses for sequence alignment.

| Orthoreovirus | Virus strain | Origin | Year of isolation | Host | GenBank accession  (σC gene) |
| --- | --- | --- | --- | --- | --- |
| N-DRV | XT18 | China | 2018 | Duck | MK749407 |
| N-DRV | HN5d | China | 2015 | Duck | KT861593 |
| N-DRV | GX-Y7 | China | 2018 | Duck | MN747010 |
| N-DRV | SY | China | 2019 | Duck | MK955827 |
| N-DRV | SD19-6201 | China | 2020 | Duck | MT829208 |
| N-DRV | TH11 | China | 2012 | Duck | KC493571 |
| N-DRV | NP03 | China | 2009 | Duck | KC312699 |
| N-DRV | SD12 | China | 2014 | Mallard wild duck | KJ879930 |
| N-MDRV | SH12 | China | 2018 | Muscovy duck | MH510251 |
| N-MDRV | DH13 | China | 2018 | Muscovy duck | MH510261 |
| N-MDRV | J18 | China | 2018 | Muscovy duck | JX478266 |
| DRV | 091 | China | 2012 | Peking duck | JX478256 |
| DRV | D2533 | Hungary | 2018 | Peking duck | MH520081 |
| MDRV | ZJ2000M | China | 2013 | Muscovy duck | KF306091 |
| MDRV | 815-12 | China | 2013 | Muscovy duck | KC508656 |
| MDRV | D1546 | Hungary | 2014 | Muscovy duck | KJ871026 |
| N-GRV | 03G | China | 2012 | Goose | JX145334 |
| GRV | D20/99 | Hungary | 2013 | Goose | KF809668 |
| ARV | 3211-V-02 | Hungary | 2016 | Chinken | KX398278 |
| ARV | S1133 | USA | 1973 | Chinken | KF741762 |
| ARV | C-98 | China | 2006 | Chinken | EF057397 |

**Table S4.** Homology analysis between LY20 strain and other strain (%).

| Virus strain | L1 | | L2 | | L3 | | M1 | | M2 | | M3 | | S1 | | σC | | S2 | | S3 | | S4 | |
| --- | --- | --- | --- | --- | --- | --- | --- | --- | --- | --- | --- | --- | --- | --- | --- | --- | --- | --- | --- | --- | --- | --- |
|  | nt | aa | nt | aa | nt | aa | nt | aa | nt | aa | nt | aa | nt | aa | nt | aa | nt | aa | nt | aa | nt | aa |
| XT18 | 98.1 | 99.7 | 99.6 | 99 | 98.8 | 99.7 | 96.1 | 90.7 | 99.6 | 99.3 | 99 | 98.3 | 98.3 | 97.7 | 98.9 | 99.1 | 98.8 | 99.6 | 98.3 | 97 | 98.1 | 95.5 |
| HN5d | 98.1 | 99.7 | 97.8 | 93.6 | 97.5 | 99.4 | 97.5 | 94.2 | 98.1 | 95.5 | 96.7 | 91.5 | 94.7 | 92 | 94.8 | 95.4 | 98.8 | 99.6 | 97.6 | 95.7 | 98.2 | 95.7 |
| GX-Y7 | 98 | 99.5 | 99.3 | 98.4 | 98.5 | 99.5 | 95.9 | 90.4 | 98.1 | 95.2 | 96.6 | 92.1 | 98 | 97.1 | 98.6 | 98.8 | 98.3 | 99.1 | 97.4 | 94.7 | 99.1 | 98 |
| SY | 98.1 | 99.6 | 97.2 | 92.4 | 97.8 | 99.3 | 96.6 | 91.6 | 97.8 | 94.9 | 97.1 | 92.3 | 98.3 | 97.3 | 98.9 | 99.1 | 98.3 | 99.6 | 97.2 | 94.4 | 98 | 95.5 |
| SD19-6201 | 96.7 | 99.3 | 96.8 | 91.2 | 97 | 99.1 | 96.2 | 90.7 | 96.6 | 91.7 | 94.7 | 86.7 | 96.2 | 93.7 | 96.6 | 96 | 87.6 | 97 | 96.9 | 94.4 | 95.5 | 87.7 |
| TH11 | 97.1 | 99.2 | 97.8 | 94.1 | 98.4 | 99.3 | 96.9 | 92.8 | 97 | 92.5 | 97.2 | 92.9 | 96.2 | 95.2 | 96.3 | 97.5 | 88.3 | 97.8 | 97.6 | 95.7 | 92.9 | 81.4 |
| NP03 | 86.6 | 98.3 | 98.2 | 94.6 | 96.8 | 98.9 | 95.8 | 89.5 | 97.3 | 93.5 | 97.4 | 89.4 | 95.7 | 93.3 | 96 | 95.7 | 88 | 97.8 | 95.9 | 92.9 | 96.6 | 90.7 |
| SD12 | 93.3 | 98.8 | 97.2 | 92.5 | 96.6 | 99 | 95.1 | 88 | 98.1 | 95.8 | 95.8 | 89.4 | 97 | 94.8 | 97.3 | 96.6 | 89.3 | 98 | 96.1 | 93.2 | 96.1 | 89.7 |
| SH12 | 98.4 | 99.6 | 97.3 | 93 | 98 | 99.2 | 97.5 | 92.5 | 96.9 | 92.2 | 97 | 92.7 | 94.8 | 91.4 | 95 | 97.2 | 98.8 | 99.3 | 97.4 | 95.4 | 97.8 | 94.7 |
| DH13 | 98.3 | 99.5 | 97.9 | 94.8 | 97.9 | 99 | 97.5 | 91.9 | 96.6 | 92.4 | 97.4 | 93.8 | 94.1 | 90.6 | 94.2 | 93.5 | 98.4 | 98.9 | 97.1 | 94.7 | 97.6 | 94.5 |
| J18 | 96.6 | 99.4 | 97 | 91.8 | 97.3 | 99 | 95.7 | 89.4 | 97.5 | 93.6 | 97.5 | 61.7 | 95.5 | 93.3 | 95.5 | 95.3 | 98.8 | 99.3 | 96.2 | 93.2 | 93.2 | 82.6 |
| 091 | 97.2 | 99 | 97.6 | 93.4 | 96.5 | 98.8 | 97.1 | 92.9 | 97.2 | 93.3 | 97.4 | 93.1 | 96.7 | 94.8 | 97 | 97.2 | 89.7 | 98.3 | 96.4 | 97.2 | 93.4 | 94.2 |
| D2533 | 86.6 | 97.3 | 87.2 | 66.1 | 80.6 | 93.6 | 73.4 | 54.5 | 88.5 | 70.1 | 86 | 66.5 | 76.5 | 69.2 | 73.7 | 78.6 | 86.3 | 92.4 | 82.9 | 69.4 | 84.7 | 59.7 |
| ZJ2000M | 86 | 97.4 | 88.2 | 68.7 | 79.9 | 93 | 80.8 | 51.6 | 67.2 | 32.3 | 86.4 | 93.2 | 33.9 | 13.7 | 49.4 | 41.9 | 89.2 | 97.5 | 69.4 | 47.8 | 85 | 60.2 |
| 815-12 | 85.8 | 97.2 | 87.8 | 67.6 | 79.7 | 92.7 | 94.2 | 86.1 | 67.1 | 32.3 | 85.8 | 94 | 33.4 | 14.5 | 49.6 | 42.2 | 87.4 | 97.1 | 69.1 | 47.6 | 84.7 | 59.9 |
| D1546 | 86.2 | 97 | 87.7 | 67.8 | 80.7 | 93.2 | 79.8 | 48.8 | 67.4 | 32.7 | 85.3 | 91.9 | 33.3 | 13.8 | 48.9 | 41.5 | 94.5 | 98 | 69.6 | 47.3 | 85 | 60.7 |
| 03G | 86.4 | 97.6 | 87.7 | 68 | 95.8 | 98.4 | 94.7 | 87.1 | 87.5 | 68.3 | 92.9 | 83.5 | 95.3 | 92.9 | 95.8 | 96 | 89.7 | 97.4 | 93.6 | 86.8 | 93.8 | 83.1 |
| D20/99 | 86.5 | 97 | 88.6 | 70.1 | 78.7 | 91.9 | 79.6 | 50.5 | 68.3 | 34.8 | 78.3 | 47.9 | 47.4 | 35.1 | 48.5 | 40.4 | 87.9 | 96.1 | 68.1 | 46.3 | 91.4 | 77.1 |
| 3211-V-02 | 78.6 | 94.8 | 76.4 | 41.6 | 71.2 | 79.7 | 73.9 | 39.6 | 75.2 | 39.8 | 70.6 | 38.5 | 44.5 | 12.1 | 39.7 | 28.9 | 79.5 | 92.8 | 68.6 | 50.1 | 80.3 | 51.9 |
| S1133 | 78.4 | 94.7 | 75.7 | 40.9 | 70.8 | 79.7 | 74.5 | 42 | 77.5 | 45.6 | 70.8 | 38.3 | 45.2 | 11.3 | 39.6 | 28.3 | 79.7 | 92.4 | 69.5 | 52.4 | 79.5 | 50.6 |
| C-98 | 78.5 | 95 | 76.3 | 41.2 | 70.8 | 79.6 | 79.6 | 42.2 | 77.8 | 46 | 70.6 | 38.1 | 45 | 11.3 | 40 | 28.6 | 79.7 | 92.6 | 69.4 | 52.2 | 79.3 | 50.1 |
